# Volume-Sensitive Anion Channels Mediate Osmosensitive Glutathione Release from Rat Thymocytes

**DOI:** 10.1371/journal.pone.0055646

**Published:** 2013-01-30

**Authors:** Ravshan Z. Sabirov, Ranokon S. Kurbannazarova, Nazira R. Melanova, Yasunobu Okada

**Affiliations:** 1 Department of Cell Physiology, National Institute for Physiological Sciences, Okazaki, Aichi, Japan; 2 Laboratory of Molecular Physiology, Institute of Bioorganic Chemistry, Academy of Sciences of the RUz, Tashkent, Uzbekistan; 3 Department of Biophysics, National University, Tashkent, Uzbekistan; Albany Medical College, United States of America

## Abstract

Glutathione (GSH) is a negatively charged tripeptide, which is a major determinant of the cellular redox state and defense against oxidative stress. It is assembled inside and degraded outside the cells and is released under various physiological and pathophysiological conditions. The GSH release mechanism is poorly understood at present. In our experiments, freshly isolated rat thymocytes were found to release GSH under normal isotonic conditions at a low rate of 0.82±0.07 attomol/cell/min and that was greatly enhanced under hypoosomotic stimulation to reach a level of 6.1±0.4 attomol/cell/min. The swelling-induced GSH release was proportional to the cell density in the suspension and was temperature-dependent with relatively low activation energy of 5.4±0.6 kcal/mol indicating a predominant diffusion mechanism of GSH translocation. The osmosensitive release of GSH was significantly inhibited by blockers of volume-sensitive outwardly rectifying (VSOR) anion channel, DCPIB and phloretin. In patch-clamp experiments, osmotic swelling activated large anionic conductance with the VSOR channel phenotype. Anion replacement studies suggested that the thymic VSOR anion channel is permeable to GSH^−^ with the permeability ratio P_GSH_/P_Cl_ of 0.32 for influx and 0.10 for efflux of GSH. The osmosensitive GSH release was trans-stimulated by SLCO/OATP substrates, probenecid, taurocholic acid and estrone sulfate, and inhibited by an SLC22A/OAT blocker, *p*-aminohippuric acid (PAH). The inhibition by PAH was additive to the effect of DCPIB or phloretin implying that PAH and DCPIB/phloretin affected separate pathways. We suggest that the VSOR anion channel constitutes a major part of the γ-glutamyl cycle in thymocytes and, in cooperation with OATP-like and OAT-like transporters, provides a pathway for the GSH efflux from osmotically swollen cells.

## Introduction

Stressed cells release their metabolic constituents as a message reporting to their neighbors about the physiological or pathophysiological cellular processes occurring inside the signal-emitter cells. ATP is one of the most recognized metabolites and is released from virtually every type of cells in the body [1]. In our previous studies, we demonstrated that electrogenic pathway mediated by activation of the maxi-anion channel contributes to the massive release of ATP from a number of cell types under a variety of stresses [2]–[7]. Osmotically and metabolically stressed cells release also other metabolites such as glutamate, which, in case of primary cultured astrocytes, exits the cells via two types of anion channels: the maxi-anion channel and the volume-sensitive outwardly rectifying (VSOR) anion channel [8]. Upon bradykinin stimulation, massive release of glutamate takes place via ROS and Ca^2+^-nanodomain-mediated VSOR anion channel activation with virtually no contribution of the maxi-anion channel [9]–[11]. Both maxi-anion and VSOR channels belong to the class of volume-regulated anion channels (VRACs) [12], [13]; and they possess relatively wide pores with the effective radii of 1.3 nm [14] and 0.62 nm [15] for the maxi-anion and VSOR channels, respectively. Thus, it is feasible that these VRACs can pass other organic anions.

Glutathione (GSH) is a tripeptide (γ-L-glutamyl-L-cysteinylglycine) bearing one net negative charge. Our molecular modeling (see [13], [16] for calculation method) yielded an effective GSH radius of 0.52–0.56 nm, implying that the molecule could be accommodated or even passed by VRACs. Therefore, we hypothesized that activation of the maxi-anion and/or VSOR channels could result in a release of this key regulator of cellular oxidation/reduction status.

GSH is a ubiquitous and most prevalent intracellular thiol tripeptide found throughout the body and involved not only in maintaining the cytosolic redox potential and defense against the oxidative stress but also in many other cellular processes including protein and nucleic acid synthesis, regulation of cell cycle, proliferation, exocrine secretion and thermotolerance [17]–[22]. In most cells, the cytosolic concentration of GSH is in the range of 1–10 mM (>98% in thiol-reduced form), whereas micromolar concentrations are found in the plasma [17], [19], [20]. The biosynthesis of GSH occurs intracellularly, whereas it is degraded exclusively outside the cells via cleavage by an ectoenzyme, γ-glutamyl transpeptidase and by dipeptidase [17]. Therefore, constant delivery of GSH and its S-conjugates to the cellular exterior is an important step for the γ-glutamyl cycle, which constitutes the cyclic process connecting between the GSH metabolism and the transport of amino acids. GSH release has been reported to be induced or enhanced by a variety of stimuli including hypoosmotic stress [23].

In the present study, we demonstrate that hypoosmotic stress induces mass release of GSH from rat thymocytes, which occurs via two main pathways: the VSOR anion channel and organic anion transporters with no detectable contribution of the maxi-anion channel.

## Materials and Methods

### Ethics statement

The experimental protocol was approved in advance by the Ethics Review Committee for Animal Experimentation of the National Institute for Physiological Sciences.

### Solutions and chemicals

The normal isotonic Ringer solution contained (in mM): 135 NaCl, 5 KCl, 2 CaCl_2_, 1 MgCl_2_, 5 Na-HEPES, 6 HEPES, and 5 glucose (pH 7.4, 290 mosmol/kg-H_2_O). Hypotonic solutions were prepared by mixing the normal Ringer solution with a HEPES-buffer solution containing (in mM): 5 KCl, 2 CaCl_2_, 1 MgCl_2_, 5 Na-HEPES, 6 HEPES, and 5 glucose (pH 7.4, 38 mosmol/kg-H_2_O). The pipette solution for whole-cell experiments contained (in mM): 125 CsCl, 2 CaCl_2_, 1 MgCl_2_, 3 Na_2_ATP, 5 HEPES (pH 7.4 adjusted with CsOH), and 10 EGTA (pCa 7.65; 275 mosmol/kg-H_2_O). The hypertonic pipette solution (320 mosmol/kg-H_2_O) was made by adding 50 mM mannitol. For measurements of glutamate or GSH permeability, the low-Cl^−^ bath solution was prepared by replacing 135 mM NaCl in the normal Ringer solution with 135 mM Na-glutamate or Na-GSH. In some experiments, for measurements of GSH permeability, the low-Cl^−^ pipette solution was prepared by replacing 100 mM CsCl in the pipette solution with 100 mM Cs-GSH.

Nicotinamide adenine dinucleotide phosphate (NADPH) and glutathione reductase were from Oriental Yeast (Tokyo, Japan), 4-[(2-butyl-6,7-dichloro-2-cyclopentyl- 2,3-dihydro-1-oxo-1*H*-inden-5-yl)oxy]-butanoic acid (DCPIB) was from TOCRIS Biosciences (Bristol, UK), and all other reagents were from Sigma-Aldrich (St. Louis, MO). GdCl_3_ was stored as a 50 mM stock solution in water and added directly to the experimental solution immediately before each experiment. Other drugs were stored as 1000-times stocks in DMSO and added to the experimental solution immediately before use. DMSO did not have any effect, when added alone at a concentration of ≤0.1%.

Osmolality of all solutions was measured using a freezing-point depression osmometer (OM802: Vogel, Kevelaer, Germany).

### Cells

Cell isolation was performed as described previously [24]–[26]. Briefly, the 6–8 weeks old rats were anaesthetized with halothane or diethyl ether and painlessly euthanized by cervical dislocation; the thymi were dissected and carefully washed with an ice-cold Ringer solution. The thymi were then minced using fine forceps and passed through a 100 µm-nylon mesh. The suspension was centrifuged at 1000 g for 5 min, the pellet was washed two times with the normal Ringer solution and resuspended in this medium at a cell density of (1–15)×10^8^ cells/ml. The cell suspension was kept on ice for ≤5 h and contained no more than 5% of damaged cells as assayed by trypan blue exclusion.

### Glutathione release assay

The bulk extracellular GSH concentration was measured by an enzymatic recycling method by reduction of 5,5′-dithiobis-2-nitrobenzoic acid (DTNB, Ellman's reagent) in yellow-colored 5-thio-2-nitrobenzoic acid (TNB) as described elsewhere [27], [28]. Briefly, the cell suspension was diluted 1:10 with the normal or hypotonic Ringer solution and incubated at 25 °C (if not indicated specifically). In some experiments, the cells were exposed to the normal Ringer solution supplemented with 500 mM mannitol. At the specified time points, the cell suspension was centrifuged at 1000 g for 10 min, and 125 µl aliquots of the supernatants were collected for a photometric assay. The aliquots were mixed with 375 µl of a cocktail containing (in mM): 133 MES (2-(morfolino) ethanesulphonic acid), 33 KH_2_PO_4_, 0.66 EDTA, 0.11 NADPH, and 0.2 DTNB (pH 6.0). The cocktail was prepared on the day of experiment and was additionally supplemented with 0.25 U/ml glutathione reductase (EC 1.6.4.2) immediately before use. The mixture was incubated in dark for 25 min at room temperature and the optical density was measured at 412 nm. The GSH concentration was calculated from a standard calibration curve obtained using the same procedure performed with pure GSH in a range from 0 to 16 µM. When required, drugs were added to the normal or hypotonic solutions to give the final concentrations as indicated. The drugs at the concentrations employed in the present study had no significant effect on the assay reaction. In some experiments, treatment with a GSH scavenger, 2-vinylpyridine (2-VP), was performed with supernatants for 60 min at room temperature. Since at the used concentration (30 mM), 2-VP had a mild inhibiting effect on the enzymatic reaction, the error caused by this effect was accounted for by calibration using oxidized glutathione (GSSG) as a substrate.

The total intracellular GSH content in 1 ml of the suspension containing 1×10^8^ cells was determined after cells were lysed with 1% Triton X-100 and the supernatants were deproteinated by treatment with 5% metaphosphoric acid and 200 mM triethanolamine [27].

### Electrophysiology

Patch electrodes were fabricated from borosilicate glass capillaries using a laser micropipette puller (P-2000, Sutter Instrument, Novato, CA) and had a tip resistance of 3–5 MΩ when filled with pipette solution. Fast and slow capacitative transients were routinely compensated for. For whole-cell recordings, the access resistance did not exceed 10 MΩ and was always compensated for by 80%. Membrane currents were measured with an EPC-9 patch-clamp system (Heka-Electronics, Lambrecht/Pfalz, Germany). The membrane potential was controlled by shifting the pipette potential (*Vp*) and is reported as *Vp* for whole-cell recordings. Currents were filtered at 1 kHz and sampled at 5–10 kHz. Data acquisition and analysis were done using Pulse+PulseFit (Heka-Electronics). Whenever the bath Cl^−^ concentration was altered, a salt bridge containing 3 M KCl in 2% agarose was used to minimize variations of the bath electrode potential. Liquid junction potentials were calculated using pCLAMP 8.1 (Axon Instruments, Foster, CA) algorithms and were corrected off-line when appropriate. The relative mobility of GSH^−^ (0.24) was determined in 10 mM water solution by conductivity measurements performed as described earlier [14]. All experiments were performed at room temperature (23−25 °C).

### Data analysis

For whole-cell macroscopic currents, the reversal potentials were either calculated by fitting instantaneous current-voltage (I–V) curves to a second-order polynomial [14] or were measured directly from the ramp I–V relationships. The permeability ratio for an organic anion X^−^ (glutamate^−^ or GSH^−^) was calculated from reversal potential shifts upon ion replacement based on the Goldman-Hodgkin-Katz (GHK) equation:
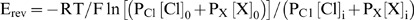
(1)


where *E_rev_* is the reversal potential; *[Cl]_o_* and *[Cl]_i_* are the Cl^−^ concentrations on the extracellular and intracellular sides, respectively; *[X]_o_* and *[X]_i_* are the concentrations of the organic anion X^−^ on the extracellular and intracellular sides, respectively (see corresponding solutions for respective experimental conditions). *P_Cl_* and *P_X_* are the permeability coefficients of Cl^−^ and organic anion X^−^, respectively.

Data were analyzed by OriginPro 7.0 (MicroCal Software, Northampton, MA). Pooled data are given as means ±SEM of observations (*n*). Statistical differences of the data were evaluated by ANOVA and the paired or unpaired Student's *t* test where appropriate, and considered significant at P<0.05.

## Results

### Swelling-induced GSH release

**Figure 1:**
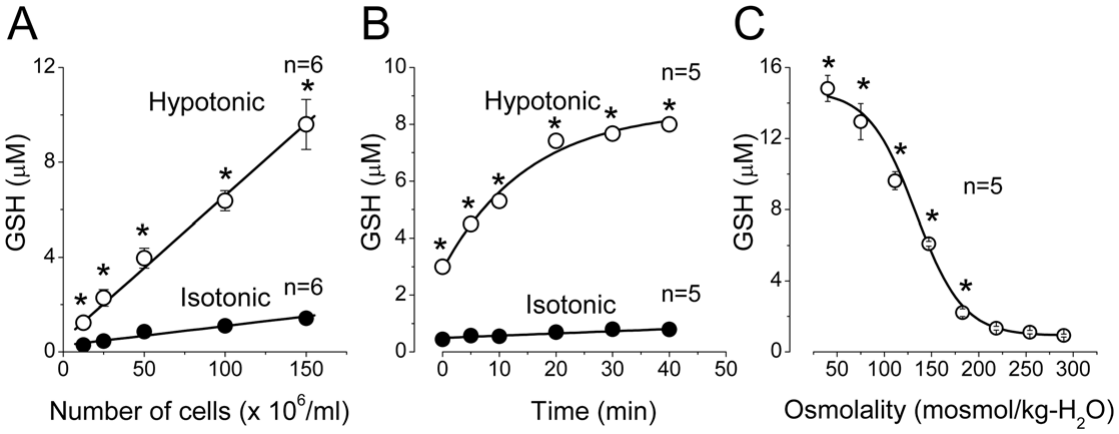
Osmosensitive release of GSH from rat thymocytes. (A) Dependence of the GSH concentration in the extracellular fluid on the number of cells in suspension under control isotonic (290 mosmol/kg-H_2_O) conditions (filled symbols) and upon hypotonic (147 mosmol/kg-H_2_O) stimulation (open symbols). The cells were incubated 10 min at 25^o^C. (B) Time course of GSH release from rat thymocytes under control isotonic conditions (filled symbols) and upon hypotonic (147 mosmol/kg-H_2_O) stimulation (open symbols). The suspension containing 1 x 10^8^ cells/ml was incubated at 25^o^C for the indicated time. (C) Osmolality dependence of GSH release from rat thymocytes. The suspension containing 1 x 10^8^ cells/ml was incubated at 25^o^C for 20 min in solutions with various tonicity. *Significantly different from the isotonic control values at P<0.05.

In normal isotonic Ringer solution (290 mosmol/kg-H_2_O), the basal release of GSH from rat thymocytes was low and totaled 0.29±0.08 µM in the suspension containing 1.25×10^7^ cells/ml and 1.42±0.09 µM (*n* = 6) in the suspension of 1.50×10^8^ cells/ml after 10-min incubation at 25 °C (Fig. 1A). When the extracellular osmolarity was increased by adding 500 mM mannitol, the basal extracellular GSH level was only slightly increased (by 29.0±2.6%, *n* = 5; P<0.05). In contrast, when cell swelling was induced by exposing to hypotonic solution (147 mosmol/kg-H_2_O), the extracellular GSH concentration drastically increased and reached the levels of 1.23±0.10 µM (*n* = 6) and 9.60±1.10 µM (*n* = 6) in the suspensions containing 1.25×10^7^ cells/ml and 1.50×10^8^ cells/ml, respectively (Fig. 1A). The GSH concentration in the extracellular medium was a linear function of the number of cells in the suspension both under normal isotonic conditions and under the hypoosmotic stress (Fig. 1A). The slope of this relationship yielded a rate of GSH release from a single cell equal to 0.82±0.07 attomol/cell/min in basal conditions and 6.1±0.4 attomol/cell/min under the hypoosmotic stress. The GSH scavenger, 2-VP, reduced the observed GSH signal by 64.3±6.3% (*n* = 4) in basal isotonic conditions and by 85.2±2.4% (*n* = 4) under the hypoosmotic stress, suggesting that glutathione is released from thymocytes predominantly in the reduced, but not oxidized, form.

The time course of GSH accumulation in the extracellular fluid was monotonic in the normal Ringer solution. However, when cells were suspended in the hypoosmotic solution, we observed an initial jump immediately after adding the cells followed by a further gradual increase in the GSH concentration which reached a steady-state level after approx. 20 min of incubation (Fig. 1B). Such biphasic kinetics of GSH release may suggest the presence of at least two different pathways with different kinetic parameters.

When the cells were incubated in solutions of different osmolality (ranging from 290 to 40 mosmol/kg-H_2_O) for 20 min, the release of GSH increased with decreasing the medium osmolality (Fig. 1C). The osmolality dependence of the GSH release could be well fitted by a sigmoidal function with half-maximal activation at 135.1±2.9 mosmol/kg-H_2_O.

**Figure 2:**
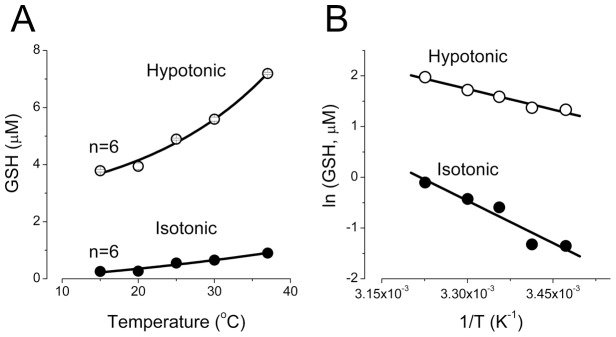
Effects of ambient temperature on the osmosensitive release of GSH from rat thymocytes. (A) The suspension containing 1×10^8^ cells/ml was incubated at indicated temperature for 10 min. All values measured in hypoosmotic conditions were significantly different from the isotonic control values at P<0.05. (B) The data from (A) are presented as an Arrhenius plot. The solid lines are linear fits with the activation energies given in the text.

Temperature of the incubation medium had a strong effect on the GSH release from thymocytes both in isotonic and hypotonic conditions. The extracellular GSH concentration gradually increased with the temperature rise from 15 °C to 37 °C (Fig. 2A). In the range from 15 °C to 37 °C, the temperature dependence of GSH release was linear in Arrhenius coordinates (Fig. 2B) with apparent activation energy of 11.1±1.8 kcal/mol and 5.4±0.6 kcal/mol for isotonic basal and hypotonic stress conditions, respectively. A relatively low level of the activation energy obtained for the swelling-induced GSH release may suggest a substantial contribution of some channel-mediated diffusional pathway to the observed release of GSH from osmotically swollen thymocytes.

Under hypotonic conditions (147 mosmol/kg-H_2_O, for 20 min), the total intracellular GSH content was found to be reduced by 56.9±1.9% (*n* = 7) at 25 °C. When 1 or 5 mM GSH was added to the extracellular solution, hypotonic stress reduced the total GSH level only by 41.0±4.9% (*n* = 4; P<0.05) at 1 mM and turned to increase by 39.0±16.3% (*n* = 5; P<0.05) at 5 mM. These results suggest that the GSH efflux from swollen thymocytes is mainly driven by its electrochemical gradient.

### Contribution of anion channels to the swelling-induced GSH release

**Figure 3:**
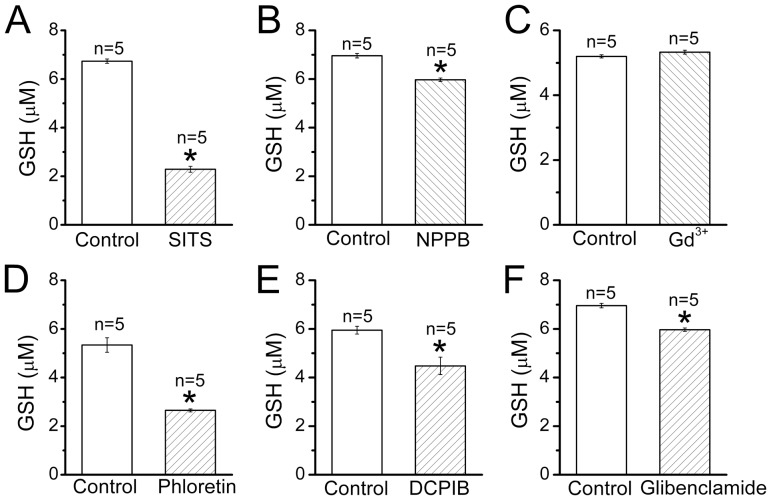
Effects of anion channel blockers on the GSH release from rat thymocytes upon hypotonic stimulation. The suspension containing 1×10^8^ cells/ml was incubated for 20 min at 25 °C in the hypoosmotic (147 mosmol/kg-H_2_O) conditions in the absence of any drug (control) or presence of 200 µM SITS (A), 200 µM NPPB (B), 50 µM Gd^3+^ (C), 200 µM phloretin (D), 20 µM DCPIB (E) and 200 µM glibenclamide (F). *Significantly different from the control values at P<0.05.

Since the molecule of GSH bears one net negative charge, we hypothesized that anion-transporting pathways may contribute to the GSH release from thymocytes. Indeed, a broad-spectrum anion transport inhibitor, SITS (200 µM), and a non-specific anion channel blocker, NPPB (200 µM), significantly suppressed not only the basal GSH release (by 97.1±4.5 and 48.8±4.6%, *n* = 5, respectively) but also the swelling-induced GSH release from rat thymocytes (Fig. 3, A and B). However, gadolinium (50 µM), a blocker of maxi-anion channels, which are expressed in many cell types [16], [29], was not effective for both basal (data not shown, *n* = 6) and swelling-induced GSH release (Fig. 3C), suggesting that this channel does not contribute to the GSH release from rat thymocytes. In contrast, phloretin (200 µM) and DCPIB (20 µM), both of which are blockers relatively specific to the VSOR anion channel [30], [31], markedly suppressed the swelling-induced release of GSH from rat thymocytes (Fig. 3, D and E), whereas phloretin-induced inhibition of the basal GSH release was less marked (by 28.2 ± 2.3%, *n* = 5). Glibenclamide (200 µM), which is known to block the VSOR anion current effectively at positive potentials but only weakly at negative potentials [32], also significantly suppressed the GSH release from swollen thymocytes (Fig. 3F). These results suggest that the VSOR anion channel serves as a major pathway for the swelling-induced GSH release from rat thymocytes. However, even most effective phloretin eliminated only approximately half of the total GSH release indicating that some other pathways may exist for GSH efflux from rat thymocytes under hypoosmotic stress.

### Contribution of organic anion transporters to the swelling-induced GSH release

**Figure 4:**
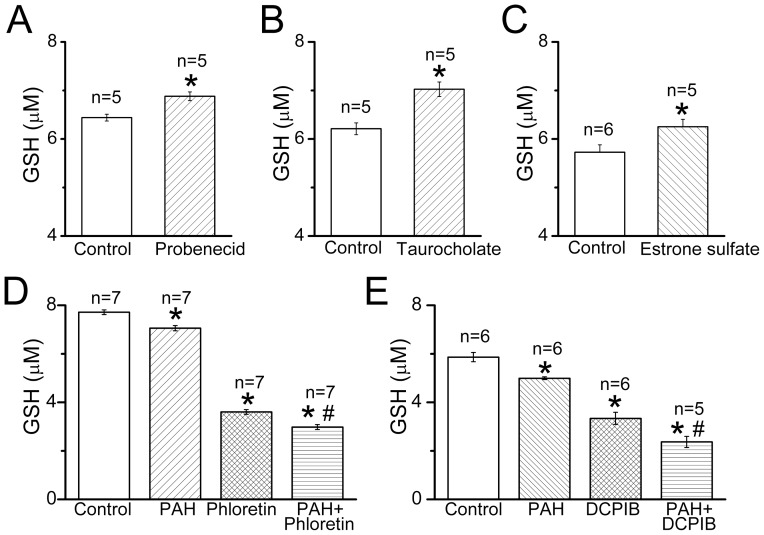
Effects of organic anion transporter substrates and inhibitors on the swelling-induced GSH release from rat thymocytes. Stimulating effects of the SLCO/OATP substrates, probenecid (A), taurocholic acid (B) and estrone sulfate (C), and a suppressing effect of the inhibitor of SLC23A/OAT transporter, *p*-aminohippuric acid (PAH), alone and in combination with VSOR blockers, phloretin (200 µM) (D) and DCPIB (20 µM) (F) on the net GSH release from rat thymocytes upon hypotonic stimulation are shown. The suspension containing 1×10^8^ cells/ml was incubated for 20 min at 25 °C in the hypoosmotic (147 mosmol/kg-H_2_O) conditions in the absence of any drug (control) or presence of the above-indicated drugs used at the concentration of 500 µM (A), 2 mM (B and C) and 1 mM (D and F). In order to minimize day-to-day variations, the results shown in (D) and (F) were obtained within one experiment performed in one day. *Significantly different from the control values at P<0.05. ^#^Significantly different from phloretin (D) or DCPIB (F) alone at P<0.05.

Several anion transporter-mediated mechanisms can be envisioned for the swelling-induced GSH release. First, the multidrug resistance-associated proteins (ABCC/MRP family) could be activated and pump GSH out of the cells. However, MK571, a specific MRP inhibitor, had no effect on the swelling-induced GSH release at 50 µM (*n* = 5, data not shown). When we tested probenecid, which is also a known inhibitor of the ABCC/MRP proteins, we observed a stimulating rather than inhibiting effect of this drug (Fig. 4A). We supposed that this action could be a result of trans-stimulation of an equilibrative organic exchanger. Indeed, taurocholic acid and estrone sulfate, which are substrates for organic anion-transporting polypeptide proteins of SLCO/OATP family produced a trans-stimulation effect similar to the probenecid effect on the swelling-induced GSH release (Fig. 4, B and C) confirming an involvement of the anion exchange mechanism in the GSH release from rat thymocytes.

An inhibitor of the sodium-dependent organic anion transport mechanism mediated by SLC22A/OAT family transporters, *p*-aminohippuric acid (PAH), produced a significant block of the swelling-induced GSH release from rat thymocytes (Fig. 4, D and F). Based on this result, we suggest that the portion of GSH release from swollen thymocytes, which was insensitive to the VSOR anion channel blockers, is likely to be mediated by organic anion transport via SLCO/OATP-like and SLC22A/OAT-like groups of membrane transporters, but not by ATPases of the ABCC/MRP type. When PAH was applied together with a VSOR anion channel blocker, phloretin (200 µM) or DCPIB (20 µM), suppression of the GSH release was more prominent (Fig. 4, D and F) than that observed with each drug applied separately. Such additivity suggests that the VSOR channel and the SLC22A/OAT-like transporter represent independent pathways for the GSH efflux from swollen thymocytes.

### GSH permeability of the swelling-activated anion conductance

**Figure 5:**
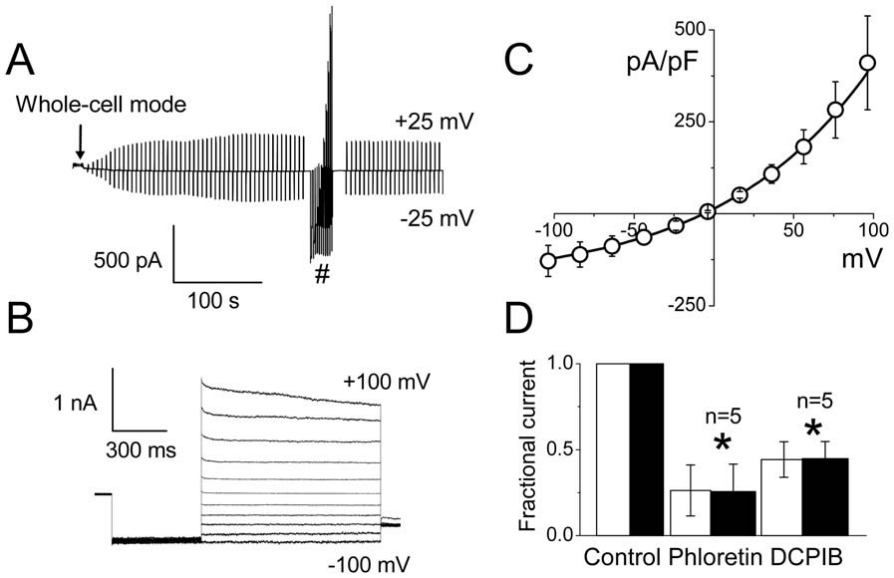
Whole-cell macroscopic currents activated by cell swelling in rat thymocytes. (A) Time course of whole-cell current activation in response to cell swelling. Currents were elicited by application of alternating pulses from 0 to ±25 mV (every 5 s). (B) Representative traces of current responses recorded at the steady-state in (A) marked with # symbol. The holding potential was 0 mV; after a pre-pulse to −100 mV (500 ms), currents were elicited by application of step pulses (1000 ms) from −100 to +100 mV in 20-mV increments. (C) Instantaneous I-V relationship measured at the beginning of test pulses from recordings similar to those shown in (B). (D) Effects of VSOR channel blockers, phloretin (200 µM) and DCPIB (10 µM), on the whole-cell macroscopic currents activated by cell swelling in rat thymocytes. Open and filled bars represent the fractional currents measured at +25 mV and −25 mV, respectively. *Significantly different from the control values at P<0.05.

In the following experiments, we tested the possibility of physical movement of the GSH molecule through the plasma membrane of swollen rat thymocytes using the patch-clamp method. As in our previous study of mouse thymocytes [24], we induced cell swelling by employing the hypertonic pipette solution (made by adding mannitol) rather than the hypotonic bath solution, since under this experimental design the whole-cell configuration was more stable and reproducible, and thereby swelling-induced macroscopic currents could be consistently observed. As shown in a representative recording, the macroscopic current developed after attaining the whole-cell configuration using the hypertonic pipette solution, and gradually increased with cell swelling reaching a steady-state level after about 3 min (Fig. 5A). The current responses to step pulses exhibited time-dependent inactivation at positive potentials larger than +80 mV (Fig. 5B). The instantaneous I–V relationship showed moderate outward rectification and reversed at −4.2±1.6 mV (Fig. 5C), which is close to the equilibrium Cl^−^ potential of −2.8 mV. The steady-state current densities at all applied voltages were close to those measured in mouse thymocytes under the same experimental conditions [24]. The macroscopic current measured at ±25 mV was markedly suppressed by the VSOR anion channel blockers, phloretin (200 µM) and DCPIB (10 µM), as shown in Fig. 5D.

**Figure 6:**
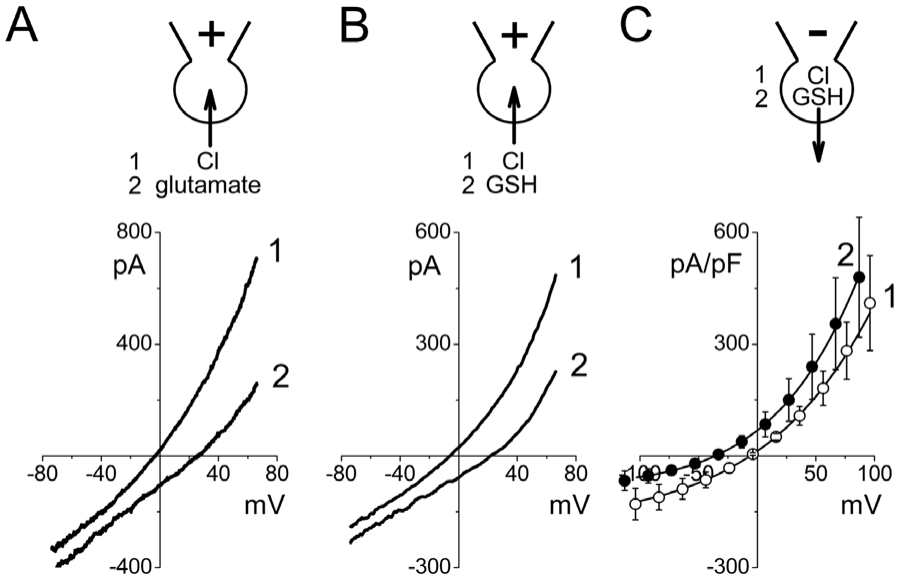
Glutamate and GSH permeability of the whole-cell currents activated by cell swelling in rat thymocytes. (A) Whole-cell current responses to ramp pulses from −70 to +70 mV before and after replacement of extracellular Cl^−^ with glutamate. The pipette solution was Cl^−^-rich (125 mM CsCl); the bath solution was normal Ringer (curve 1) or low-Cl^−^ Ringer solution in which 135 mM NaCl was replaced with 135 mM Na-glutamate (curve 2). (B) Whole-cell current responses to ramp pulses from −70 to +70 mV before and after replacement of extracellular Cl^−^ with GSH. The pipette solution was Cl^−^-rich (125 mM CsCl); the bath solution was the normal Ringer (curve 1) or low-Cl^−^ Ringer solution in which 135 mM NaCl was replaced with 135 mM Na-GSH (curve 2). (C) Instantaneous whole-cell I–V relationships in response to step pulses in 20 mV increments. The pipette solution was Cl^−^-rich (curve 1: 125 mM CsCl) or GSH^−^-rich (curve 2: 100 mM Cs-GSH plus 25 mM CsCl). The bath solution was the normal Ringer solution. Averaged data of 6 experiments performed in each configuration are plotted.

In the following series of experiments, we monitored the reversal potential changes upon ion substitution. After full activation of the swelling-induced macroscopic conductance, the whole-cell current responses to ramp pulses from −70 to +70 mV exhibited moderate outward rectification (Fig. 6A, trace 1) comparable to that observed in instantaneous I–V relationships from step-pulses (Fig. 5C). The ramp I–V curves reversed at −5.8±0.5 mV (*n* = 5) when the bath was filled with the normal Ringer solution. The reversal potential shifted to the value of +25.4±5.9 mV (*n* = 4) upon equimolar replacement of 135 mM Cl^−^ with glutamate^−^ in the bath solution (Fig. 6A, trace 2) indicating anion selectivity of the whole-cell macroscopic conductance with the permeability ratio (evaluated by Eqn. (1)) of P_glutamate_/P_Cl_ = 0.24±0.08. Next, we performed similar experiments with equimolar replacement of 135 mM Cl^−^ with GSH^−^ and obtained a shift of the reversal potential from −4.7±0.5 mV (*n* = 6) to the value of +20.7±2.5 mV (*n* = 5) (Fig. 6B), yielding the permeability ratio of P_GSH_/P_Cl_ = 0.32±0.04. It should be noted that in these experiments, the direction of the GSH flux was inward, which is opposite to the outward flow of GSH observed in the swelling-induced GSH release experiments. Therefore, we next tested the glutathione permeability by equimolarly replacing 100 mM of Cl^−^ ions in the pipette solution with GSH^−^. This maneuver produced a leftward shift of the reversal potential from −5.1±1.8 mV (*n* = 6) to the value of −34.8±1.1 mV (*n* = 6), yielding the permeability ratio of P_GSH_/P_Cl_ = 0.10±0.03. Based on these results, we conclude that the VSOR anion channels activated on the plasma membrane of osmotically swollen rat thymocytes have substantial permeability to the negatively charged GSH molecule providing a physical path for its swelling-induced efflux. A lower permeability ratio for the GSH efflux compared to its influx may reflect the outwardly rectifying nature of the VSOR anion channel.

## Discussion

Delivery of GSH to the cellular milieu is an important step in the γ-glutamyl cycle. Various stimuli have been reported to augment the GSH release, including oxidative stress in Calu-3 airway epithelial cells [33], divalent cation-free [34] or low extracellular Ca^2+^ medium [35] in cultured rat astrocytes, glutamate treatment in retinal cell cultures [28], NMDA and kainate [36] and high K^+^-induced depolarization [37] in rat brain slices, cerebral ischemia [38]–[40] and administration of ammonia [41] in microdialyzed rat brain, norepinephrine administration in canulated leghorn carotid artery, hepatic and portal veins and bile duct [42], apoptotic stimuli [43], [44] in epithelial [45]–[50] and lymphoid [51]–[54] cells.

In the perfused whole-liver model, hypotonic exposure was found to increase hepatic GSH output in the effluent, and the main component was interpreted as an enhancement in the electrogenic efflux due to increased potassium conductance and membrane hyperpolarization [23]. The present paper represents a first report of the osmotic swelling-stimulated GSH release at the cellular level. Moderate GSH efflux from thymocytes observed under basal conditions and its profound increase by cell volume expansion would suggest that extracellular GSH plays an important role in the thymic environment.

The mechanism of GSH exit from cells is poorly understood at present. Since GSH is a negatively charged ion, it is conceivable that an anion channel may mediate its electrogenic translocation through the plasma membrane. Indeed, the broad-spectrum anion transport inhibitors, SITS and NPPB, significantly suppressed the swelling-induced GSH efflux (Fig. 3). Previously, the cystic fibrosis transmembrane conductance regulator (CFTR), a cAMP-activated anion channel, which has a pore wide enough to accommodate GSH [55], was shown to be permeable to GSH based on the data obtained by patch-clamp [56] and proteoliposome experiments [57]. Consistently, in the airway epithelial cells, a high level of GSH delivery to the extracellular space was greatly diminished in cells derived from ΔF508 homozygous cystic fibrosis (CF) patients and could be restored by repleting the cells with normal CFTR protein [58]. However, the observation that deficient GSH release of the CF epithelium can be restored also by a CFTR-unrelated synthetic Cl^−^ channel-forming peptide, N-K4-M2GlyR, raises a possibility that CFTR acts as a regulator of another GSH-exporting pathway in the airway epithelium [59].

The results of the present study provide strong biophysical, pharmacological and electrophysiological evidence that the VSOR anion channel represents one of major pathways for the GSH efflux induced by osmotic cell swelling in rat thymocytes. First, low activation energy for the swelling-induced GSH release is an indicative of the channel-mediated GSH^−^ transport (Fig. 2). Second, VSOR anion channel blockers, DCPIB and phloretin, eliminated one-third to half of the total GSH release from osmotically stressed cells (Fig. 3, D and E). Third, the ion replacement patch-clamp experiments revealed a substantial permeability of the thymic VSOR anion channel to GSH^−^ anions (Fig. 6). The permeability for GSH^−^ was asymmetric in accord with the rectifying nature of the VSOR anion channel. The released GSH was predominantly in the reduced, but not oxidized, form. This fact is in agreement with the concept that the efflux is mediated by the VSOR anion channel, the pore size of which is too narrow to pass the exceedingly large oxidized dimmer, GSSG.

In our recent study, we found that the single VSOR channels recorded from swollen mouse thymocytes [24] had a unitary current-voltage relationship very similar to that observed earlier in human epithelial Intestine 407 cells [60], bovine endothelial cells [61], mouse mammary C127 cells [62], mouse ventricular myocytes [63], mouse cortical neurons [64], mouse cortical astrocytes [8] and other cell types [12], [13], [65]. Using the reported unitary amplitude of ∼1 pA at −60 mV (assumed to be close to the resting membrane potential of thymocyte) and P_GSH_/P_Cl_ = 0.1, we can estimate the rate of GSH transport through a single open VSOR channel to be around 6×10^5^ GSH molecule/s. The GSH efflux of 6.1 attomol/cell/min measured for rat thymocytes under osmotic stress corresponds to the transport rate of approx. 0.6×10^5^ GSH molecule/s. This would mean that even a single constantly open VSOR channel could provide a level of GSH release comparable or even higher than that observed in the experiments. The steady-state whole-cell current of −500 pA at −60 mV (Fig. 5B) suggests that a single thymocyte may express several hundreds (∼500 in this particular cell) of VSOR anion channels. Our results would suggest that only a small fraction of them will be active during the GSH release experiment, supposedly because the cells will be restoring their volume by the regulatory volume decrease (RVD) mechanism, which is very prominent in these cells [25], [26]. In addition, swelling-induced membrane depolarization might also diminish the electrochemical driving force for GSH efflux.

In case of mice, thymocytes functionally express not only VSOR but also maxi-anion channels [24]. Hence, it is surprising that we were unable to detect any contribution of this channel to the swelling-induced GSH release in the present study. One explanation could be that the maxi-anion channel is expressed differentially in different species. Indeed, our patch-clamp study failed to detect any maxi-anion channel activity in thymocytes isolated from rats (Kurbannazarova, Kurganov and Sabirov, unpublished observation).

DCPIB and phloretin suppressed swelling-induced GSH release from thymocytes by 25−40% (Figs. 3E and 4E) and 50−53% (Figs. 3D and 4D), respectively, whereas SITS, a broad-spectrum anion transport inhibitor, suppressed it by around 97% (Fig. 3A). DCPIB is a blocker most specific to the VSOR channel [31], whereas phloretin is known to not only block more effectively the VSOR channel compared to other anion channels [30] but also inhibit aquaporins [66] and glucose transporters [67]. Therefore, it is likely that the swelling-induced GSH release from thymocytes is largely (up to 25−40%) mediated by the VSOR anion channel. Some additional phloretin- and/or SITS-sensitive pathways permeable to GSH may also be involved in the GSH release. However, an involvement of aquaporins seems unlikely, because the aquaporin pore is very tight and allows transport of only water and some small nonelectrolytes, but hardly would pass bulky GSH^−^ anion.

To date, numerous transporters capable of translocating large organic anions across the plasma membrane have been characterized, and GSH could be a substrate for some of them. Thus far, most of the authors considered two major types of transporters as putative GSH releasing pathways. First is the family of multidrug resistance-associated proteins (ABCC/MRP), which are implicated in high constitutive GSH efflux in the liver [19], [20], [68] and brain astrocytes [21], [69]. MRP1-overexpression studies with BHK [46] and HEK293 [50] cells as well as siRNA-mediated MRP1 gene silencing in Jurkat cells [54] supported the notion of MRP-mediated apoptotic GSH release. In our present study, however, an MRP inhibitor, probenecid, produced stimulation rather than inhibition of the swelling-induced GSH release from rat thymocytes arguing against the contribution of this pathway in immature thymic lymphocytes. Also, a selective MRP inhibitor, MK571, was found to have no effect on the swelling-induced GSH release at 50 µM in the present study. This result is in contrast to the previous observations that MK571 stimulated and inhibited the GSH release from astrocytes at 1 and 50 µM, respectively [70], and stimulated the GSH release from MRP1-overexpressing leukemia cells at 50 µM [71].

The second commonly considered GSH-transporting pathway is the organic anion transporters of SLCO/OATP family. In the liver, the OATPs are presumably located on the sinusoidal membrane and responsible for the GSH efflux from hepatocytes to the bloodstream [72], [73]. In Jurkat T-lymphocytes, OATPs but not MRPs were responsible for the apoptogenic GSH efflux [43], [51]. We now show that in thymocytes, the swelling-induced GSH release is trans-stimulated by three OATP substrates, probenecid, taurocholic acid and estrone sulfate (Fig. 4, A–D), a result consistent with the significant contribution of the SLCO/OATP-like pathway. An inhibiting effect of PAH, a blocker of the SLC22A/OAT group of anion transporting proteins, would suggest a contribution of the sodium-dependent transport mechanism in the GSH release from thymocytes (Fig. 4, D and E). The SLC22A/OAT-like route is separate from the VSOR anion channel as evidenced by additivity of the effects of the blockers of these two pathways (Fig. 4, D and E). It is noted that Na^+^-dependent GSH transport was reported in human cerebrovascular endothelial HCEC cells [74] and in retinal cell cultures [28], although it was not evident in the apoptotic GSH efflux from Jurkat cell [51].

**Figure 7:**
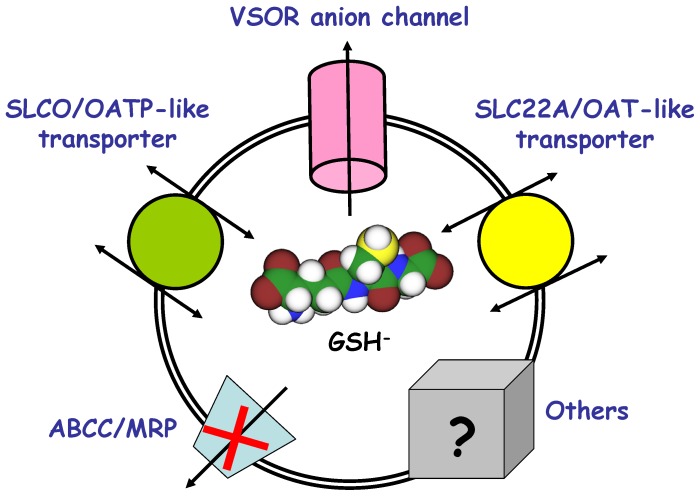
Hypothetical pathways that contribute to the GSH release from swollen thymocytes. The scheme depicts three main pathways of the osmotic swelling-induced GSH release revealed in the present study: VSOR anion channel (sensitive to DCPIB and phloretin), SLC22A/OAT-like transporter (sensitive to PAH), and SLCO/OATP-like transporter (stimulated by the substrates such as probenecid, taurocholate and estrone sulfate). Significant contribution of the MRP protein (sensitive to MK571 and probenecid) was not detected in the present study. The grey box denotes other possible routes for GSH exit from swollen thymocytes.

In conclusion, we suggest that the VSOR anion channel constitutes a major part of the γ-glutamyl cycle in thymocytes and, in cooperation with OATP-like and OAT-like transporters, provides a pathway for the GSH efflux from osmotically swollen cells, as schematically depicted in Fig. 7.
